# Liver Function-Related Indicators and Risk of Gallstone Diseases—A Multicenter Study and a Systematic Review and Meta-Analysis

**DOI:** 10.1155/2024/9097892

**Published:** 2024-08-24

**Authors:** Shiyi Li, Pei Zhu, Fangyuan Chen, Wenqian Yu, Linjun Xie, Jing Xia, Peng Jiao, Ping Cui, Chi Zhang, Ye Bai, Guoheng Jiang, Hongyu Li, Yanmei Lou, Guangcan Li, Xuefeng Shan, Xin Wang

**Affiliations:** ^1^ West China School of Public Health Sichuan University, South Renmin Road, Wuhou District, Chengdu 610041, China; ^2^ Department of Vaccine Clinical Research Institute Mianyang City Center for Disease Control and Prevention, Mianyang, Sichuan Province, China; ^3^ Military Casualty Management Department General Hospital of the Western War Zone of the Chinese People's Liberation Army, Chengdu 610036, China; ^4^ Department of Health Management Jining No 1 People's Hospital Jining, Shandong, China; ^5^ Department of Public Health Jining Medical University, Jining 272067, China; ^6^ Department of Prevention Tianjin Medical University Cancer Institute and Hospital, Tianjin 300060, China; ^7^ Gene Diagnosis Center Bethune First Hospital Jilin University, Changchun 130000, China; ^8^ Department of Health Management Beijing Xiaotangshan Hospital, Beijing 102211, China; ^9^ Department of Pharmacy The People's Hospital of Kaizhou District, Chongqing, No. 8, Ankang Road, Hanfeng Street, Kaizhou District, Chongqing 405400, China; ^10^ Department of Pharmacy Bishan Hospital of Chongqing Medical University, Chongqing 402760, China

**Keywords:** gallstone disease, liver function-related indicators, multicenter cross-sectional study, systematic review and meta-analysis

## Abstract

**Purpose of the study:** We aim to examine the association between liver function-related indicators and gallstone disease (GSD) risk.

**Study design:** The subjects who participated in the China Multicenter Physical Examination Cohort (CMPEC) were enrolled. Relative odds ratios (ORs) with 95% CIs and standardized mean differences (SMDs) were applied to investigate the effect of liver function-related indicators and GSD risk. Moreover, a systematic review and meta-analysis were conducted until July 2021. Additionally, the results in the CMPEC and the systematic review and meta-analysis were combined by meta-analysis. Finally, the results were validated by a cohort study of the UK Biobank (UKB).

**Results and conclusions:** Totally, 369,931 subjects in CMPEC were included in the study. A total of 28 publications were incorporated into the systematic review and meta-analysis. The pooled analysis suggested that aspartate aminotransferase (AST), alanine aminotransferase (ALT), alkaline phosphatase (ALP), total protein (TP), and low albumin (ALB) were positively associated with the risk of GSD. Meanwhile, GSD present to have higher AST, ALT, gamma-glutamyl transferase (GGT), total bilirubin (TBil), globulin (G), and ALP levels and relatively lower TP and ALB levels than the healthy participants. These results were consistent when stratified by the study design, geographic background, and study quality. Only the association between ALP and GSD risk was validated in the UKB cohort. This study suggests liver function indicators were associated with GSD risk. The results may provide the basis for exploring the etiology of GSD and may help clinicians identify high-risk subjects.

**Trial Registration:** PROSPERO (CRD42020179076).

## 1. Introduction

Gallstone disease (GSD) is a common digestive disease. The frequency of GSD varies widely between countries due to differences in genetics, environment, lifestyle, etc. In Europe, about 20% of adults harbor GSD [1]. In comparison, the prevalence of GSD in Chinese adults is about 3%–11% [2]. The prevalence of GSD in women is higher than in men [3].

During the lifetime of GSD patients, more than a fifth will experience biliary symptoms or complications and require surgical management [4]. It is reported that over 50,000 cholecystectomies are performed each year in the United Kingdom. While in the United States, cholecystectomies reach approximately 800,000 and consume nearly 6.0 billion dollars annually, which inflicts heavy health burdens and economic costs [5, 6]. Furthermore, it is reported that GSD will increase the risk of diabetes [7], tumor [8–10], and all-cause mortality [11, 12], which will bring severe disease and economic burden to patients. Thus, it is essential to study the cause of GSD for disease prevention and control.

Previous studies suggested liver function indicators were associated with GSD risk [3, 13, 14]. However, due to the relatively smaller sample size, the study design variations, and the geographic background, the reported results were inconsistent [15–17].

China Multicenter Physical Examination Cohort (CMPEC) is a multicenter study that enrolled the subjects who participated in the health examination in nine hospitals in Tianjin, Beijing, Chongqing, Sichuan, and Shandong provinces of China between 2015 and 2020, which incorporated about 1.2 million participants [18].

Therefore, we first conduct a cross-sectional study through CMPEC to find the association between liver function-related indicators and GSD risk. Secondly, we conduct a systematic review and meta-analysis aiming to confirm the association. And then, combined the abovementioned results by meta-analysis to investigate the associations between liver function-related indicators and the GSD risk. Ultimately, the present study tried to validate these associations using the cohort study of the UK Biobank (UKB) [19].

## 2. Materials and Methods

### 2.1. Study Population

CMPEC consisted of nine hospitals. The present study included the participants who underwent health check-ups in Chongqing Kaizhou District People's Hospital, First Affiliated Hospital of Chongqing Medical University, Beijing Xiaotangshan Hospital, Tianjin Medical University Cancer Institute and Hospital, and Chongqing Qianjiang Central Hospital in China between January 2015 and May 2020. Participants aged ≥ 18 underwent abdominal ultrasonography, had laboratory results of liver function-related indicators, and signed informed consent were included. The Ethics Committee of West China Fourth Hospital and West China School of Public Health, Sichuan University approved the study. (Gwll2021055).

### 2.2. Physical Examination

The physical examination of the subjects in CMPEC was administered and recorded by trained investigators. The demographic data were collected, including age and sex, and anthropometric data were measured when undressed, including height, weight, and waist circumference (WC). Body mass index (BMI) (kg/m^2^) is calculated by dividing the weight (kilograms) by the square of height (meters). Blood pressure monitoring is administered in a resting state. The participant takes a sitting position, and elbows are naturally placed approximately parallel to the heart to detect the systolic blood pressure (SBP) and diastolic blood pressure (DBP) of the right brachial artery.

### 2.3. Laboratory Examinations

Approximately 10 ml of peripheral venous blood was collected from each subject after an overnight fast of at least 8 h. Serum parameters, including FPG, TC, triglycerides, aspartate aminotransferase (AST), alanine aminotransferase (ALT), total bilirubin (TBil), gamma-glutamyl transferase (GGT), alkaline phosphatase (ALP), total protein (TP), globulin (G), albumin (ALB) were monitored using an automated biochemical analyzer according to standard protocols. This study divided liver function-related indicators into two or three groups according to the clinical diagnosis: AST (U/L): >42, and 0–42; ATL (U/L): >40, and 0–40; ALP (U/L): >150, and 0–150; GGT (U/L): >50, and 0–50; TBil (umol/L): >20, and 0–20; TP (g/L): >85, 60–85, <60; ALB (g/L): >55, 30–55, <30; G (g/L): >35, 23–35, <23.

### 2.4. Ultrasonography and Definitions

Professional sonographers performed the abdominal ultrasound for the liver, gallbladder, bile duct, pancreas, spleen, and kidney. The ultrasonographic images of GSD are as follows: one or more strong echoes with acoustic shadows in the gallbladder cavity, extrahepatic bile duct, or intrahepatic bile duct, which can move with the change of body position [20]. Cholecystectomy was defined as a history of gallbladder removal operation and/or no gallbladder visible at ultrasonography due to gallstones [21]. GSD was diagnosed as the presence of gallstones and/or cholecystectomy [22].

### 2.5. Systematic Review and Meta-Analysis

The meta-analysis has been registered at PROSPERO (CRD42020179076). We systematically searched Embase and Pubmed databases to identify relevant English publications until July 2021. In addition, we searched the China National Knowledge Infrastructure (CNKI) database and Wanfang Data Knowledge Service Platform to obtain qualified Chinese Studies. The keywords include “gallstones” OR “gallstone disease” OR “cholelithiasis” AND “alanine aminotransferase” OR “aspartate aminotransferase” OR “alkaline phosphatase” OR “bilirubin” OR “serum protein” OR “gamma-glutamyl transferase” OR “risk factors” OR “influencing factors.” Two authors independently performed a literature search, article selection, data extraction, and quality assessment. All inconsistent data were discussed and resolved by the corresponding authors. In addition, we searched the references of eligible studies to include additional studies.

Inclusion criteria were as follows: (1) observational studies, including cross-sectional, case-control, or cohort studies; (2) investigating the relationship between liver function-related indicators and GSD risk in the general population; (3) provide relative risk (RR), odds ratio (OR), or hazard ratio (HR) with a 95% confidence interval (CI), or provide sufficient data to calculate these risk estimates; (4) provide standard mean difference (SMD) with 95% CI values between GSD patients and health control or provide sufficient data to calculate the SMD and 95% CI. When multiple studies focus on the same population, we only included the most comprehensive study with the largest sample size.

Data were extracted from a predesigned table, including the first author, publishing year, study period, exposure factors, geographical background, study design, sample size, the number of male and female participants, and the average level of liver function-related indicators in GSD and non-GSD groups. We extracted the most fully adjusted estimates when multiple adjusted models were used in the study. As recommendations, the quality of the included studies was assessed according to the Newcastle–Ottawa Scale (NOS) [23]. A score of ≥ 7 was deemed high quality, while a score of < 7 was regarded as low quality.

### 2.6. UKB

The UKB is a large prospective cohort study established to investigate various diseases' genetic and lifestyle determinants, having the characteristics of large sample size, rich variables, and high data quality. In the UKB, a total of 500,000 volunteers between the ages of 40 and 69 were enrolled from the United Kingdom, which includes demographic characteristics such as age and gender; serological test indicators such as FPG and TC; behavior and lifestyle indicators such as smoke and drink; body measurement indicators, such as height and weight; disease information, such as diabetes and hypertension; abdominal ultrasound examination, such as GSD and nonalcoholic fatty liver disease (NAFLD) [19]. The application number of this research is Project50538.

### 2.7. Statistical Analysis

Continuous variables were described as mean ± SD, and differences between GSD and control group were compared by Student's *t*-test. Categorical variables were expressed in frequency (percentage), and differences between groups were compared by the chi-square test or Wilcoxon's rank-sum test.

For each center of the CMPEC, the relationship between liver function-related indicators and GSD risk was assessed by multivariable logistic regression analysis adjusting for confounding factors, including age, gender, BMI, kidney stones, fatty liver, high blood pressure, blood lipid levels (TC and TG), FBG, blood pressure levels (SBP and DBP). In addition, we conducted subgroup analyses by age (< 40, vs. 40–60, vs. > 60 years) and gender (male vs. female). For the systematic review and meta-analysis, when the included studies provide the OR and 95% CIs, meta-analysis for the pooled OR was assessed to examine the associations. For the included studies that offer the mean levels of liver function-related indicators and SD, meta-analysis for continuous variables was conducted to obtain standard mean difference (SMD). The *I*^2^ was used for quantifying the amount of variance. The DerSimonian and Laird random-effects model was used for the systematic review and meta-analysis when *I*^2^ ≥ 50%. Otherwise, the fix-effect model was applied. The study design, geographic background, and NOS score also performed subgroup analyses. In addition, sensitivity analysis by sequentially omitting each study was conducted to assess the stability of the pooled results. Potential publication bias was evaluated by a significant funnel plot asymmetry and Egger's test.

For the UKB, the relationship between liver function-related indicators and GSD risk was assessed by multivariable Cox regression analysis, adjusting for confounding factors, including age, gender, BMI, WHR, Type I diabetes, Type 2 diabetes, nonalcoholic fatty liver, cirrhosis, hypertension, dyslipidemia, blood lipid levels (TC, triglycerides, HDL, and LDL), kidney stones, chronic kidney disease, cystatin C, apolipoprotein A, apolipoprotein B, C-reactive protein, and uric acid.

All statistical analyses were performed by using SPSS 26.0 (IBM, USA). Meta-analysis was performed by Stata 16 (Stata, College Station, TX). The statistically significant level was two-tailed and was set at *p* < 0.05.

## 3. Results

### 3.1. Baseline Characteristics of Recruited Subjects in CMPEC

A total of 369,931 participants in the CMPEC were included, and 173,050 of them were males (46.8%); the average age was 42.35 years. For different centers, the GSD prevalence ranged from 3.48% in Chongqing Qianjiang Central Hospital to 8.35% in the People's Hospital of Kaizhou District of Chongqing. Meta-analysis suggested that the pooled GSD prevalence was 5.68% (95% CI: 4.20%, 7.64%). The demographic and clinical characteristics are listed in Table 1.

Compared with the non-GSD group, the GSD group had higher s BMI, DBP, SBP, FBG, TC, TG, and LDL-C levels and a higher proportion of fatty liver disease, hypertension, kidney stones, and gallbladder polyps than the non-GSD group. While the HDL-C, ALB, and TP levels were relatively lower.

### 3.2. The Associations Between Liver Function-Related Indicators and GSD Risk in the CMPEC

Multivariable logistic regression analysis showed the associations between AST, ALT, TBil, and GSD risk were inconsistent between different centers in the CMPEC. The results are listed in Table S2. After pooling results from multicenter data of CMPEC using meta-analysis, the results showed a higher level of AST (pooled OR = 1.12, 95% CI: 1.06–1.18, *p* < 0.001), ALP (pooled OR = 1.73, 95% CI: 1.40−2.14, *p* < 0.001), low level of TP (pooled OR = 1.33, 95% CI: 1.02−1.73, *p* = 0.007) was positively associated with GSD risk. In contrast, high levels of ALB (pooled OR = 0.54, 95% CI: 0.48−0.60, *p* < 0.001) were negatively related to the risk of GSD. The associations between higher levels of ALT (pooled OR = 1.06, 95% CI: 0.98−1.15, *p* = 0.131), TBil (pooled OR = 1.02, 95% CI: 0.93−1.12, *p* = 0.694), GGT (pooled OR = 1.14, 95% CI: 0.94, 1.39, *p* = 0.181), high level of G (pooled OR = 0.68, 95% CI: 0.10−4.68, *p* = 0.698), and low level of G (pooled OR = 0.49, 95% CI: 0.10−2.32, *p* = 0.367) and GSD did not reach the significant level (Table 2). The mean ALT (SMD = 0.06, 95% CI: 0.03−0.10, *p* = 0.01), ALP (SMD = 0.26, 95% CI: 0.13−0.39, *p* < 0.001), and GGT (SMD = 0.11, 95% CI: 0.07−0.15, *p* < 0.001) levels were higher in GSD-group than that control group (Table 2). These results were not consistent in different gender and age groups.

### 3.3. Systematic Review and Meta-Analysis

#### 3.3.1. Characteristics of the Included Studies

**Figure 1:**
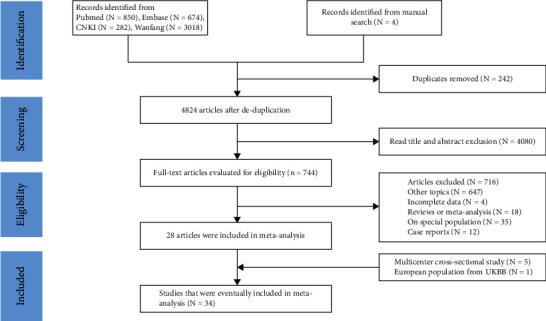
Flow chart for systematic review and meta-analysis.

We initially found 5066 records from Embase, Pubmed, CNKI, and Wanfang databases. Based on the inclusion and exclusion criteria, we finally included 23 articles, which included approximately 355,854 (including 14,707 GSD cases and 341,147 controls) participants. The flow chart is shown in Figure 1. Table S3 reports the characteristics of the included publications. Of these studies, eight were published in English [3, 13–17, 22, 24], and 20 were published in Chinese [25–44]. A total of 16 [3, 13, 14, 16] [17, 22, 25–28, 32, 34, 35, 37, 42, 44] were cross-sectional studies and 12 [15, 24, 29–31, 33, 36, 38–41, 43] were case-control studies. Most of the participants were from China (*N* = 26), followed by Korea (*N* = 1) [16], and India (*N* = 1) [15], respectively. The quality of included studies was evaluated according to NOS: 15 studies scored ≥7 [13–15, 17, 24–26, 28, 29, 31, 37, 38, 42–44], with a minimum score of 5 (*N* = 4) [33, 35, 36, 45] and a maximum score of 8 (*N* = 2) [14, 31].

#### 3.3.2. The Associations Between Liver Function-Related Indicators and GSD Risk in the Systematic Review and Meta-Analysis

The systematic review and meta-analysis showed that AST (pooled OR = 1.30, 95% CI: 1.01–1.68, *p* = 0.04), ALT (pooled OR = 1.16, 95% CI: 1.04–1.29, *p* = 0.006), and ALP (pooled OR = 1.49, 95% CI: 1.12–1.96, *p* = 0.006) were positively associated with the risk of GSD in previous studies (Figure S1).

After adding the results of the CMPEC, results suggested AST (pooled OR = 1.19, 95% CI = 1.08 ~ 1.31, *p* < 0.001), ALT (pooled OR = 1.07, 95% CI:1.04~1.11, *p* < 0.001), ALP (pooled OR = 1.35, 95% CI:1.02~1.77, *p* = 0.34), and low TP (pooled OR = 1.32, 95% CI:1.08~1.61, *p* = 0.007) were positively related to the risk of GSD, while high ALB (pooled OR = 0.54, 95% CI:0.48~0.60, *p* < 0.001) were negatively related to the risk of GSD. As shown in Table 3, the mean of all indicators' levels was higher in the GSD group than that in the control group (*p* < 0.001), except for TP (SMD = −0.13, 95% CI: −0.20~−0.06, *p* < 0.001) and ALB (SMD = −0.14, 95% CI: −0.15~ −0.12, *p* < 0.001).

Subgroup analysis also indicated that the associations were generally consistent when stratified by study design, NOS score, and geographic background. (Table 4) In some indicators, there was heterogeneity among the included studies, and meta-regression showed that the study design and geographic background might be the sources of heterogeneity (Table 4). Sensitivity analysis (Figure S2) shows that when a single study is omitted, some indicators results have changed, but most indicators results have no noticeable change. In addition, Egger's test showed no significant difference in publication bias (the funnel charts are shown in Figure S3).

#### 3.3.3. Baseline Characteristics of Recruited Subjects in UKB

A total of 462,903 participants in the UKB were included, and 210,741 of them were males (45.5%), and the average age was 56.76 years. The median follow-up time was 150 months, and the interquartile interval was 20 months. During the follow-up, a total of 15,071 participants (2.66%) developed GSD, and 6842 of them were males (45.4%) (Table S1). The multivariable Cox analysis in the UKB showed that ALP (HR = 1.09, 95% CI: 1.07–1.11, *p* < 0.001) was positively associated with GSD risk. In contrast, AST (HR = 0.95, 95% CI: 0.94–0.977, *p* < 0.001) was negatively related to the risk of GSD. (Table 5).

## 4. Discussion

In our study, we first conduct a cross-sectional study through CMPEC to find the associations between liver function-related indicators and GSD risk. After that, we conduct a systematic review and meta-analysis to confirm these associations. And then, we combined the abovementioned results by meta-analysis to get a relatively definitive conclusion. Ultimately, we verify these associations based on the subjects in the UKB cohort.

Results for the CMPEC and the systematic review and meta-analysis found a high level of AST, ALT, and ALP were positively associated with GSD risk, and high TP and ALB levels were negatively associated with GSD risk. While in the UKB, only the association between ALP and GSD was validated. Besides, AST was negatively associated with GSD risk in the UKB.

Our study reveals a compelling positive correlation between ALP and the risk of GSD, suggesting that higher levels of ALP may significantly contribute to an increased susceptibility to developing GSD. The mechanism remains unclear, possibly because when hepatic cells are injured, ALP, a biomarker of hepatobiliary disease and bone resorption, is commonly present in liver and bile duct cells. This may increase the pressure in the bile duct and reduce bile excretion, thereby increasing the risk of GSD [46]. Therefore, the level of ALP can be used as an effective indicator for clinical diagnosis and prediction of gallstone onset.

ALB, as a crucial constituent of TP, is synthesized by liver parenchymal cells at a rate contingent upon colloid osmotic pressure and dietary protein intake, thereby capable of reflecting the liver functional reserve [47]. Our research indicates that high levels of TP and ALB are negatively correlated with the risk of GSD. However, this association was not validated by the UKB, and more studies are warranted to further confirm these results.

AST and ALT are markers that reflect hepatocyte damage and enter the bloodstream during hepatocyte necrosis or increased hepatocyte membrane permeability, with increased enzyme activity in the blood [48, 49]. The results of the multicenter study and the systematic review and meta-analysis showed that AST and ALT were positively associated with the risk of GSD. While in the UKB, AST was negatively associated with GSD risk, and ALT was not significantly associated with GSD. Cross-sectional studies in Chinese populations concluded that AST was not significantly associated with GSD [50]. One study [17] also showed no association between ALT and the risk of GSD. That may be because ALT is more sensitive or specific to the liver, resulting in unstable analysis results of ALT. The elevation of AST may also be considered secondary to nonhepatic causes, but the exact mechanism of AST remains unclear. In our study, the meta-analysis results of the association between AST and GSD were inconsistent with those obtained by UKB. In the future, we need more multicenter-cohort studies to further verify the results.

### 4.1. Limitations

There are some limitations to our study. Firstly, only cross-sectional data of the CMPEC is available for this part, and the findings may be influenced by potential confounders and reverse causality. Further prospective studies are subsequently needed to verify the association between liver function-related indicators and GSD risk. Secondly, most of the studies included in the meta-analysis were from China, which may be subject to some bias, due to the unavailability of the original data of the included studies, most of the studies could not be meta-analyzed for OR values. To minimize this bias, we performed a meta-analysis of SMD values for all studies (Table 3). Finally, in this study, we only studied gallstones and did not include choledocholithiasis or other gallbladder diseases, and there is a need for conducting further studies on other gallbladder diseases in the future.

## 5. Conclusion

In conclusion, our findings show that ALP, low TP, and low ALB are risk factors for GSD, which can provide a basis for identifying the high-risk groups of GSD and exploring the pathogenesis of GSD. When the serological indexes of the liver function of patients are abnormal, clinical attention should be paid to them. Besides taking effective treatment and preventive measures, health publicity and education should be strengthened to improve self-care awareness to reduce the incidence of GSD. In addition, monitoring serological indexes related to liver function can provide a basis for identifying high-risk groups, early predicting, and improving prognosis.

## Figures and Tables

**Table 1:** Baseline characteristics of recruited subjects according to gallstone disease status.

	**First Affiliated Hospital of Chongqing Medical University Jinshan Hospital (** **N** = 121071**)**				**The People's Hospital of Kaizhou District of Chongqing (** **N** = 103427**)**				**Beijing Xiaotangshan Hospital (** **N** = 60945**)**				**Tianjin Medical University Cancer Institute and Hospital (** **N** = 10236**)**				**Chongqing Qianjiang Central Hospital (** **N** = 74252**)**			
	**GSD**	**Non-GSD**	**t/chi-square**	**p**	**GSD**	**Non-GSD**	**t/chi-square**	**p**	**GSD**	**Non-GSD**	**t/chi-square**	**p**	**GSD**	**Non-GSD**	**t/chi-square**	**p**	**GSD**	**Non-GSD**	**t/chi-square**	**p**
Total	9547	111524			8633	94794			3319	57626			474	9762			2586	71666		
Age	51.98 ± 12.73	41.89 ± 12.97	−74.21	<0.001	50.88 ± 11.06	43.43 ± 11.98	−59.55	<0.001	5.63 ± 14.10	43.58 ± 13.32	−48.03	<0.001	55.43 ± 13.21	42.64 ± 13.21	−20.59	<0.001	51.26 ± 12.77	38.09 ± 14.01	−51.34	<0.001
Gender (male)	5085 (53.3%)	65144 (58.4%)	95.74	<0.001	4370 (50.6%)	57138 (60.3%)	306.1	<0.001	2020 (60.9%)	35288 (61.2%)	0.19	0.667	227 (47.9%)	4230 (43.3%)	3.82	0.51	1172 (45.3%)	37352 (52.1%)	46.21	<0.001
Body mass index	24.52 ± 3.06	23.28 ± 3.16	−37.74	<0.001	25.23 ± 3.26	24.05 ± 3.45	−32.1	<0.001	26.08 ± 3.27	25.13 ± 3.54	−16.15	<0.001	—	—	—	—	24.55 ± 3.16	23.58 ± 3.31	−15.31	<0.001
DBP (mmHg)	78.13 ± 11.98	74.63 ± 11.42	−27.51	<0.001	80.12 ± 12.58	77.62 ± 12.27	−17.72	<0.001	76.40 ± 10.40	74.65 ± 10.69	−9.41	<0.001	80.68 ± 12.25	76.50 ± 11.76	−7.54	<0.001	79.03 ± 12.04	76.09 ± 10.80	−12.25	<0.001
SBP (mmHg)	129.65 ± 19.29	122.98 ± 17.13	−32.73	<0.001	128.90 ± 19.00	123.55 ± 17.54	−25.18	<0.001	125.92 ± 16.58	120.39 ± 16.25	−18.71	<0.001	139.15 ± 20.72	127.35 ± 18.83	−12.16	<0.001	130.17 ± 19.35	124 ± 16.40	−16	<0.001
FBG (mmol/L)	5.83 ± 1.58	5.42 ± 1.14	−25.09	<0.001	5.96 ± 1.70	5.56 ± 1.30	−21.27	<0.001	5.87 ± 1.45	5.5 ± 1.21	−14.41	<0.001	—	—	—	—	5.67 ± 1.71	5.27 ± 1.17	−11.91	<0.001
TC (mmol/L)	5.04 ± 0.99	4.91 ± 0.94	−12.27	<0.001	5.11 ± 1.01	5.02 ± 0.98	−7.94	<0.001	4.93 ± 0.98	4.82 ± 0.93	−6.57	<0.001	5.57 ± 1.03	5.34 ± 1.01	−4.81	<0.001	5.03 ± 0.97	4.88 ± 0.94	−8.03	<0.001
TG (mmol/L)	1.92 ± 1.86	1.60 ± 1.42	−16.46	<0.001	1.99 ± 1.66	1.73 ± 1.48	−13.95	<0.001	1.70 ± 1.24	1.61 ± 1.36	−4.16	<0.001	1.56 ± 0.94	1.32 ± 0.88	−5.84	<0.001	2.10 ± 1.67	1.75 ± 1.44	−12.18	<0.001
LDL-C (mmol/L)	3.06 ± 0.83	2.95 ± 0.81	−12.91	<0.001	2.69 ± 0.69	2.63 ± 0.68	−7.37	<0.001	3.08 ± 0.80	3.00 ± 0.78	−5.96	<0.001	—	—	—	—	2.70 ± 0.75	2.63 ± 0.73	−4.84	<0.001
HDL-C(mmol/L)	1.36 ± 0.33	1.41 ± 0.34	13.32	<0.001	1.37 ± 0.41	1.40 ± 00.42	8.26	<0.001	1.31 ± 0.32	1.35 ± 0.33	6.02	<0.001	—	—	—	—	1.34 ± 0.33	1.39 ± 0.35	7.53	<0.001
AST (U/L)	23.84 ± 10.94	23.26 ± 14.30	−3.83	<0.001	26.49 ± 13.22	26.14 ± 16.25	−1.97	0.049	22.69 ± 11.14	21.92 ± 10.10	−4.25	<0.001	20.66 ± 15.02	18.77 ± 8.93	−2.72	0.007	25.22 ± 11.92	23.83 ± 12.66	−5.79	<0.001
ALT (U/L)	27.88 ± 20.95	26.83 ± 24.10	−4.65	<0.001	29.18 ± 22.38	28.33 ± 25.93	−3.32	0.001	24.79 ± 19.66	24.32 ± 20.10	−1.31	0.191	31.34 ± 25.10	26.86 ± 22.60	−3.81	<0.001	27.78 ± 22.38	24.96 ± 21.12	−6.31	<0.001
Tbil (umol/L)	13.91 ± 5.51	13.69 ± 5.36	−3.81	<0.001	15.31 ± 6.06	15.10 ± 5.97	−2.96	0.003	—	—	—	—	16.83 ± 5.64	17.03 ± 5.55	0.78	0.435	14.26 ± 5.73	14.37 ± 6.12	1.005	0.315
ALP	—	—	—	—	91.07 ± 34.30	86.20 ± 26.21	−12.84	<0.001	—	—	—	—	109.41 ± 35.65	100.55 ± 30.83	−6.07	<0.001	77.51 ± 28.45	73.37 ± 26.56	−7.76	<0.001
GGT	39.51 ± 102.31	33.29 ± 41.31	−5.9	<0.001	41.87 ± 47.99	37.74 ± 60.73	−7.62	<0.001	—	—	—	—	—	—	—	—	37.89 ± 58.70	31.12 ± 48.08	−5.8	<0.001
Alb	46.35 ± 2.71	47.11 ± 2.74	26.13	<0.001	75.45 ± 3.91	75.58 ± 3.93	19.04	<0.001	—	—	—	—	48.25 ± 2.31	48.92 ± 2.41	5.84	<0.001	—	—	—	—
TP	75.89 ± 4.57	76.14 ± 4.44	5.06	<0.001	75.45 ± 3.91	75.58 ± 3.93	2.9	0.004	—	—	—	—	76.02 ± 4.48	76.50 ± 4.38	2.29	0.022	—	—	—	—
G	29.54 ± 4.28	29.03 ± 4.08	−11.34	<0.001	30.26 ± 3.31	29.85 ± 3.18	−11.07	<0.001	—	—	—	—	27.77 ± 3.82	27.58 ± 3.48	−1.16	0.246	28.32 ± 4.79	27.50 ± 4.66	−8.8	<0.001
Fatty liver disease	3982 (41.7%)	29145 (26.1%)	1073.5	<0.001	3617 (41.9%)	26696 (28.2%)	720.48	<0.001	1593 (48.0%)	21305 (37.0%)	162.6	<0.001	258 (54.4%)	2993 (30.7%)	117.86	<0.001	1092 (42.2%)	8451 (11.8%)	2064	<0.001
Kidney stones	527 (5.5%)	4815 (4.3%)	30.16	<0.001	315 (3.6%)	3302 (3.5%)	0.64	0.423	254 (7.7%)	1876 (3.3%)	179.9	<0.001	20 (4.2%)	222 (2.3%)	7.41	0.006	71 (2.7%)	717 (1.0%)	72.39	<0.001
Hypertension	3481 (36.5%)	22323 (20.0%)	1418.2	<0.001	2785 (32.3%)	20869 (22.0%)	470.79	<0.001	1503 (45.3%)	15107 (26.2%)	575.6	<0.001	239 (50.4%)	2562 (26.2%)	132.94	<0.001	761 (29.4%)	11433 (16.0%)	330.2	<0.001
Gallbladder polyps	245 (2.6%)	9817 (8.8%)	448.84	<0.001	219 (2.5%)	9625 (10.2%)	533.03	<0.001	257 (7.7%)	3923 (6.8%)	4.3	0.038	22 (4.6%)	453 (4.6%)	0	0.999	—	—	—	—

*Note:* Data are presented as the mean ± SD or n (%).

Abbreviations: Alb, albumin; ALP, alkaline phosphatase; ALT, alanine aminotransferase; AST, aspartate aminotransferase; DBP, diastolic blood pressure; FBG, fasting blood glucose; G, globulin; GGT, gamma-glutamyl transferase; HDL, high-density lipoprotein cholesterol; LDL, low-density lipoprotein cholesterol; SBP, systolic blood pressure; Tbil, total bilirubin; TC, total cholesterol; TG, triglycerides; TP, total protein.

**Table 2:** Pooled analysis of the association between liver function-related indicators and gallstone disease risk in the multicenter study.

		**Odd ratio (high vs. low)**			**Standardized mean difference (SMD)**		
		**OR (95% CI)**	**p** ** value**	***P* for interaction**	**SMD (95% CI)**	**p** ** value**	**p** ** for interaction**
AST	Total	1.12 (1.06~1.18)	<0.001		−0.07 (−0.34~0.21)	0.628	
Gender	Male	1.02 (0.96~1.10)	0.488	<0.001	−0.01 (−0.03~0.03)	0.936	<0.001
	Female	1.71 (1.52~1.92)	<0.001		0.22 (0.18~0.26)	<0.001	
Age	<40	1.13 (0.93~1.38)	0.475	0.001	0.03 (−0.02~0.09)	0.242	0.506
	40–60	1.12 (1.01~1.24)	0.005		0.01 (−0.04~0.05)	0.787	
	>60	0.98 (0.81~1.18)	0.843		−0.01 (−0.06~0.04)	0.650	
ALT	Total	1.06 (0.98~1.45)	0.131		0.06 (0.03~0.10)	0.001	
Gender	Male	0.97 (0.89~1.06)	0.545	<0.001	−0.01 (−0.04~0.04)	0.848	<0.001
	Female	1.86 (1.63~2.13)	<0.001		0.29 (0.25~0.33)	<0.001	
Age	<40	1.27 (1.19~1.36)	<0.001	0.159	0.09 (0.05~0.12)	<0.001	0.012
	40–60	1.29 (1.24~1.35)	<0.001		0.12 (0.09~0.15)	<0.001	
	>60	1.17 (1.06~1.30)	0.645		0.05 (0.02~0.09)	0.001	
Tbil	Total	0.89 (0.64~1.25)	0.694		−0.07 (−0.15~0.02)	0.128	
Gender	Male	1.14 (1.05~1.24)	0.007	<0.001	0.07 (0.02~0.11)	0.004	0.131
	Female	0.99 (0.83~1.18)	0.895		0.02 (−0.03~0.06)	0.452	
Age	<40	0.93 (0.85~1.01)	0.083	<0.001	−0.02 (−0.07~0.03)	0.448	0.382
	40–60	1.14 (0.98~1.31)	0.084		0.03 (−0.02~0.07)	0.271	
	>60	1.01 (0.87~1.16)	0.938		0.02 (−0.03~0.07)	0.429	
ALP	Total	1.73 (1.40~2.14)	<0.001		0.26 (0.13~0.39)	<0.001	
Gender	Male	1.28 (1.05~1.57)	0.102	<0.001	0.06 (0.01~0.11)	0.011	<0.001
	Female	2.23 (1.90~2.62)	<0.001		0.27 (0.23~0.32)	<0.001	
Age	<40	1.40 (0.93~2.08)	0.768	0.024	0.01 (−0.05~0.06)	0.911	0.015
	40–60	1.44 (1.22~1.71)	<0.001		0.11 (0.06~0.15)	<0.001	
	>60	1.10 (0.98~1.23)	0.180		0.08 (0.03~0.14)	0.002	
GGT	Total	1.14 (0.94~1.39)	0.181		0.11 (0.07~0.15)	<0.001	
Gender	Male	1.23 (1.09~1.39)	0.001	<0.001	0.12 (0.06~0.19)	<0.001	0.001
	Female	1.55 (0.87~2.79)	0.139		0.27 (0.21~0.33)	<0.001	
Age	<40	1.34 (1.02~1.80)	0.037	<0.001	0.19 (0.14~0.24)	<0.001	<0.001
	40–60	1.16 (1.06~1.33)	0.047		0.09 (0.05, 0.13)	<0.001	
	>60	0.94 (0.72~1.22)	0.629		0.01 (−0.05, 0.05)	0.897	

TP							
(H VS M)	Total	0.99 (0.86~1.13)	0.678				
Gender	Male	0.88 (0.74~1.06)	0.113	0.688			
	Female	1.18 (0.97~1.44)	0.151				
Age	<40	1.26 (1.00~1.58)	0.930	0.988			
	40–60	1.28 (1.05~1.56)	0.219				
	>60	1.24 (0.89~1.72)	0.128				
(L VS M)	Total	1.33 (1.02~1.73)	0.007				
Gender	Male	1.60 (1.07~2.41)	0.023	0.041			
	Female	1.02 (0.72~1.44)	0.914				
Age	<40	1.15 (0.21~6.39)	0.873	0.749			
	40–60	1.01 (0.78~1.31)	0.944				
	>60	0.87 (0.56~1.37)	0.428				

ALB							
(H VS M)	Total	0.54 (0.48~0.60)	<0.001				
Gender	Male	0.54 (0.47~0.62)	<0.001	0.197			
	Female	0.65 (0.51~0.81)	<0.001				
Age	<40	0.79 (0.67~0.94)	0.007	0.022			
	40–60	1.01 (0.84~1.21)	0.909				
	>60	1.34 (0.91~1.98)	0.140				
(L VS M)	Total	1.65 (0.88~3.08)	0.117				
Gender	Male	1.51 (0.66~3.45)	0.325	0.616			
	Female	1.72 (1.24~2.39)	0.001				
Age	<40	1.12 (0.35~3.55)	0.848	0.710			
	40–60	1.22 (0.67~2.22)	0.524				
	>60	0.91 (0.50~1.67)	0.770				

G							
(H VS M)	Total	0.68 (0.10~4.68)	0.698				
Gender	Male	0.50 (0.06~3.98)	0.511	<0.001			
	Female	1.31 (1.18~1.45)	<0.001				
Age	<40	1.17 (0.84~1.61)	0.355	0.929			
	40–60	1.23 (1.12~1.34)	<0.001				
	>60	1.25 (1.11~1.40)	<0.001				
(L vs. M)	Total	0.49 (0.10~2.32)	0.367				
Gender	Male	0.41 (0.05~3.32)	0.406	<0.001			
	Female	0.88 (0.74~1.05)	0.002				
Age	<40	0.79 (0.69~0.91)	0.001	0.029			
	40–60	0.91 (0.71~1.16)	0.425				
	>60	1.03 (0.90~1.19)	0.660				

Abbreviations: Alb, albumin; ALP, alkaline phosphatase; ALT, alanine aminotransferase; AST, aspartate aminotransferase; G, globulin; GGT, gamma-glutamyl transferase; Tbil, total bilirubin; TP, total protein.

**Table:** (a) Meta-analysis of OR values for studies

**Risk factors**	**N**	**Heterogeneity**		**Selection of model**	**Pooled analysis**			**Egger's test**	
		**I** ^2^ ** (%)**	**p** ** value**		**OR**	**95% CI**	**p** ** vaule**	**T** ** vaule**	**p** ** vaule**
AST	12	51.9	0.019	Random	1.19	1.08~1.31	<0.001	1.92	0.084
ALT	12	37.5	0.091	Fix	1.07	1.04~1.11	<0.001	2.10	0.062
GGT	6	92.2	<0.001	Random	1.12	0.97~1.31	0.128	−1.03	0.363
TBil	6	61.6	0.012	Random	1.04	0.96~1.13	0.339	0.96	0.391
ALP	6	89.2	<0.001	Random	1.35	1.02~1.77	0.034	0.15	0.887
TP (H vs. M)	3	61.9	0.073	Random	0.96	0.76~1.20	0.678	2.07	0.286
TP (L vs. M)	3	17.4	0.298	Fix	1.32	1.08~1.61	0.007	−1.33	0.410
ALB (H vs. M)	3	0	0.875	Fix	0.54	0.48~0.60	<0.001	2.33	0.258
ALB (L vs. M)	2	52.6	0.146	Random	1.65	0.88~3.10	0.117		
G (H vs. M)	4	99.9	<0.001	Random	0.68	0.10~4.68	0.698	0.2	0.862
G (L vs. M)	4	46.5	0.132	Fix	0.49	0.10~2.32	0.367	0.67	0.571

**Table:** (b) Meta-analysis of SMD values for studies

**Risk factors**	**N**	**Heterogeneity**		**Selection of model**	**Pooled analysis**			**Egger's test**	
		**I** ^2^ ** (%)**	**p** ** value**		**SMD**	**95% CI**	**p** ** vaule**	**T** ** vaule**	**p** ** vaule**
AST	24	99.2	<0.001	Random	0.57	0.43~0.72	<0.001	2.18	0.040
ALT	26	98.7	<0.001	Random	0.70	0.59~0.81	<0.001	4.39	<0.001
GGT	11	99.4	<0.001	Random	1.25	1.06~1.44	<0.001	2.89	0.018
TBil	16	98.8	<0.001	Random	0.72	0.56~0.88	<0.001	3.32	0.005
ALP	10	97.9	<0.001	Random	0.64	0.50~0.77	<0.001	2.07	0.072
TP	7	86.6	<0.001	Random	−0.13	−0.20~ -0.06	<0.001	−2.84	0.036
G	6	47.4	0.09	Fix	0.13	0.12~0.15	<0.001	−0.72	0.512
ALB	8	96.9	<0.001	Random	−0.14	−0.15~ -0.12	<0.001	−0.63	0.549

Abbreviations: Alb, albumin; ALP, alkaline phosphatase; ALT, alanine aminotransferase; AST, aspartate aminotransferase; G, globulin; GGT, gamma-glutamyl transferase; H vs. M, high vs. middle; L vs. M, low vs. middle; Tbil, total bilirubin; TP, total protein.

**Table 4:** Meta-subgroup analysis.

	**Standardized mean difference**					**Odd ratio (high vs. low)**				
	**K**	**SMD (95% CI)**	**p**	**I** ^2^ ** (%)**	**p** ** for interaction**	**K**	**OR (95% CI)**	**p**	**I** ^2^	**p** ** for interaction**
AST	24	0.57 (0.43~0.72)	<0.001	99.2		12	1.19 (1.08~1.31)	<0.001	51.9	
Study_design										
CS	14	0.23 (0.08~0.41)	0.003	99.5	0.002	10	1.19 (1.08~1.30)	<0.001	53.4	0.801
CC	10	1.16 (0.60~1.73)	<0.001	97.3		2	1.03 (0.35~3.03)	0.953	71.4	
Geographical background										
China	22	0.59 (0.44~0.75)	<0.001	99.3	<0.001	10	1.15 (1.05~1.26)	0.003	46.0	0.081
Korea	1	0.77 (0.44~1.11)	<0.001	0		1	1.51 (1.17~1.95)	0.002	0	
India	1	0.04(-0.07~0.14)	0.482	0		1	1.71 (0.86~3.40)	0.125	0	
NOS score										
≥7	15	0.37 (0.17~0.58)	<0.001	99.4	<0.001	10	1.12 (1.06~1.19)	<0.001	4.4	0.036
<7	9	0.94 (0.68~1.18)	<0.001	98.4		2	1.80 (1.16~2.80)	0.009	61.1	
ALT	26	0.70 (0.59~0.81)	<0.001	98.7		12	1.07 (1.04~1.11)	<0.001	37.5	
Study_design										
CS	15	0.37 (0.26~0.48)	<0.001	98.7	0.001	10	1.10 (1.04~1.16)	0.001	42.0	0.875
CC	11	1.41 (0.82~1.99)	<0.001	98.7		2	1.17 (0.55~2.46)	0.689	48.1	
Geographical background										
China	24	0.75 (0.63~0.86)	<0.001	98.8	<0.001	10	1.07 (1.02~1.12)	0.003	20.2	0.043
Korea	1	0.03(-0.07~0.13)	0.535	0		1	1.35 (1.10~1.66)	0.005	0	
India	1	0.36 (0.10~0.61)	0.006	0		1	1.66 (0.82~3.34)	0.160	0	
NOS score										
≥7	16	0.63 (0.48~0.77)	<0.001	98.9	0.023	10	1.07 (1.03~1.10)	<0.001	27.37	0.034
<7	10	0.96 (0.71~1.21)	<0.001	98.5		2	1.33 (1.10~1.61)	0.003	0	
GGT	8	1.79 (1.52~2.05)	<0.001	99.5		5	1.11 (0.93~1.32)	0.490	93.8	
Study_design										
CS	7	0.74 (0.55~0.93)	<0.001	99.5	0.026	4	1.11 (0.93~1.33)	0.263	95.3	0.955
CC	1	2.26 (0.93~3.60)	0.001	97.0		1	1.13 (0.57~2.27)	0.724	0	
Geographical background										
China	10	1.41 (1.21~1.61)	<0.001	99.4	<0.001	4	1.14 (0.95~1.38)	0.165	95.0	0.455
Korea	1	0.03(-0.07~0.14)	0.499	0		1	0.96 (0.74~1.24)	0.730	0	
India	—	—	—	—		0	—	—	—	
NOS score										
≥7	5	1.65 (1.35~1.95)	<0.001	99.7	0.049	4	1.14 (0.95~1.38)	0.165	95.0	0.455
<7	6	1.12 (0.67~1.57)	<0.001	98.6		1	0.96 (0.74~1.24)	0.730	0	
Tbil	16	0.72 (0.56~0.88	<0.001	98~9		6	1.04 (0.96~1.13)	0.339	66.1	
Study_design										
CS	9	1.89 (1.11~2.27)	<0.001	98.6	0.001	5	1.04 (0.95~1.13)	0.398	69.9	0.234
CC	7	0.27 (0.12~0.41)	<0.001	99.0		1	1.76 (0.74~4.20)	0.201	0	
Geographical background										
China	15	0.69 (0.53~0.86)	<0.001	98.9	0.014	5	1.04 (0.95~1.13)	0.398	69.9	0.234
Korea	0	—	—	—		0	—	—	—	
India	1	1.19 (0.83~1.55)	0.094	0		1	1.76 (0.74~4.20)	0.201	0	
NOS score										
≥7	8	0.71 (0.53~0.89)	<0.001	99.0	0.233	6	1.04 (0.96~1.13)	0.339	66.1	—
<7	8	1.08 (0.50~1.66)	<0.001	98.8		0	—	—	—	
ALP	10	0.64 (0.50~0.77)	<0.001	97.9		6	1.35 (1.02~1.77)	0.034	89.2	
Study_design										
CS	7	0.33 (0.23~0.44)	<0.001	96.9	0.014	6	1.35 (1.02~1.77)	0.034	89.2	—
CC	3	1.81 (0.64~2.98)	0.002	96.8		0	—	—	—	
Geographical background										
China	9	0.71 (0.57~0.85)	<0.001	98.1	<0.001	5	1.45 (1.09~1.94)	0.011	90.5	0.039
Korea	1	0.15 (0.05~0.25)	0.004	—		1	0.81 (0.51~1.30)	0.390	0	
India	—	—	—	—		0	—	—	—	
NOS score										
≥7	6	1.02 (0.78~1.26)	<0.001	98.6	<0.001	5	1.45 (1.09~1.94)	0.011	90.5	0.039
<7	4	0.27 (0.14~0.40)	<0.001	83.8		1	1.23 (0.77, 1.97)	0.390	0	
TP	7	-0.13(-0.20~ -0.06)	<0.001	86.6		—	—	—	—	
Study_design										
CS	4	-0.07(-0.12~ -0.02)	0.007	83.3	0.006	—	—	—	—	—
CC	3	-0.41(-0.90~0.07)	0.091	86.8		—	—	—	—	
Geographical background										
China	7	-0.13(-0.20~ -0.06)	<0.001	86.6	—	—	—	—	—	—
Korea	—	—	—	—		—	—	—	—	
India	—	—	—	—		—	—	—	—	
NOS score										
≥7	6	-0.07(-0.12~ -0.03)	0.003	73.8	<0.001	—	—	—	—	—
<7	1	-0.96(-1.31~ -0.61)	<0.001	—		—	—	—	—	
ALB	8	-0.23(-0.37~ -0.09)	0.002	96.9						
Study_design										
CS	4	-0.24(-0.42~ -0.06)	0.262	98.5	0.819	—	—	—	—	—
CC	4	-0.23(-0.64~0.17)	0.008	86.5		—	—	—	—	
Geographical background										
China	8	-0.23(-0.37~ -0.09)	0.002	96.9	—	—	—	—	—	—
Korea	—	—	—	—		—	—	—	—	
India	—	—	—	—		—	—	—	—	
NOS score										
≥7	6	-0.18(-0.33~ -0.02)	0.023	97.6	0.149	—	—	—	—	—
<7	2	-0.49(-1.36~0.38)	0.273	93.4		—	—	—	—	
G	6	0.13 (0.12~0.15)	<0.001	47.0						
Study_design										
CS	4	0.13 (0.10~0.16)	<0.001		0.353	—	—	—	—	—
CC	2	0.04(-0.17~0.24)	0.736			—	—	—	—	
Geographical background										
China	6	0.13 (0.12~0.15)	<0.001	47.0	—	—	—	—	—	—
Korea	—	—	—	—		—	—	—	—	
India	—	—	—	—		—	—	—	—	
NOS score										
≥7	6	0.13 (0.12~0.15)	<0.001	47.0	—	—	—	—	—	—
<7	0	—	—	—		—	—	—	—	

Abbreviations: Alb, albumin; ALP, alkaline phosphatase; ALT, alanine aminotransferase; AST, aspartate aminotransferase; CC, case control; CS, cross sectional; G, globulin; GGT, gamma-glutamyl transferase; Tbil, total bilirubin; TP, total protein.

**Table 5:** The association between liver function-related indicators and GSD risk in the UKB.

**Factor**	**HR (95% CI)**	**p**
Tbil	1.0003 (0.9840–1.0169)	0.9715
AST	0.9543 (0.9390–0.9698)	<0.001
ALT	0.9937 (0.9767–1.0110)	0.4733
ALP	1.0903 (1.0721–1.1087)	<0.001

Abbreviations: ALP, alkaline phosphatase; ALT, alanine aminotransferase; AST, aspartate aminotransferase; Tbil, total bilirubin.

## Data Availability

The authors have nothing to report.

## Supporting Information

Supporting Information 1 Supplementary Table S1. Demographics of the UKB sample.

Supporting Information 2 Supplementary Table S2. The association between liver function-related indicators and GSD risk in each of the five hospitals.

Supporting Information 3 Supplementary Table S3. Characteristics of the included publications of meta-analysis.

Supporting Information 4 Supplementary Figure S1. Forest plot of previous studies in meta-analysis.

Supporting Information 5 Supplementary Figure S2. Sensitivity analysis of meta-analysis.

Supporting Information 6 Supplementary Figure S3. Funnel chart of meta-analysis.
