# Supplementary material for: Liver Function-Related Indicators and Risk of Gallstone Diseases—A Multicenter Study and a Systematic Review and Meta-Analysis
Source: Gastroenterol Res Pract. 2024 Aug 24;2024:9097892. doi: 10.1155/2024/9097892 (PMC11366059; doi:10.1155/2024/9097892)
Supplement: Supporting Information 1 — Supplementary Table S1. Demographics of the UKB sample. [file 9097892.f1.docx]

| **Supplementary Table 1. Demographics of the UK Biobank sample** |
| --- |

| Characteristic | Percent (n) or Mean (SD) |
| --- | --- |
| Age | 56.76 (8.03) |
| Sex |  |
| Female | 54.47% (252162) |
| Male | 45.53% (210741) |
| BMI (kg/m2) | 27.38 (4.78) |
| 18.5-24.9 (normal weight) | 32.82% (151006) |
| ＜ 18.5 (low weight) | 5.31% (2443) |
| 25.0-29.9 (overweight) | 42.53% (195643) |
| 30.0-34.9 (obese I) | 17.35% (79798) |
| 35.0-39.9 (obese II) | 4.90% (22520) |
| ＞ 40.0 (obese III) | 1.88% (8630) |
| WHR | 0.87 (0.09) |
| TC (mmol/L) | 5.69 (1.14) |
| TG (mmol/L) | 1.74 (1.03) |
| T2DM | 1.82% (8427) |
| Hypertension | 7.67% (35517) |
| Dyslipidemia | 3.37% (15580) |
| FLD | 0.21% (994) |
| KSD | 0.68% (3127) |
| ApoA (g/L) | 1.54 (0.27) |
| ApoB (g/L) | 1.03 (0.24) |
| Glucose (mmol/L) | 5.12 (1.24) |
| UA (μmol/L) | 309.26 (80.48) |
| totalBilirubin (μmol/L) | 9.13 (4.42) |
| AST (U/L) | 26.22 (10.61) |
| ALT (U/L) | 23.52 (14.10) |
| ALP (U/L) | 83.48 (26.12) |
