# Supplementary material for: Liver Function-Related Indicators and Risk of Gallstone Diseases—A Multicenter Study and a Systematic Review and Meta-Analysis
Source: Gastroenterol Res Pract. 2024 Aug 24;2024:9097892. doi: 10.1155/2024/9097892 (PMC11366059; doi:10.1155/2024/9097892)
Supplement: Supporting Information 2 — Supplementary Table S2. The association between liver function-related indicators and GSD risk in each of the five hospitals. [file 9097892.f2.docx]

**Supplementary Table 2. The association between liver function-related indicators and GSD risk in each of the five hospitals.**

|  | **First affiliated Hospital of Chongqing Medical University** | | **The People’s Hospital of Kaizhou District of Chongqing** | | **Beijing Xiaotangshan Hospital** | | **Tianjin Medical University Cancer Institute and Hospital** | | **Chongqing Qianjiang Central Hospital** | |
| --- | --- | --- | --- | --- | --- | --- | --- | --- | --- | --- |
|  | **OR (95%CI)** | **P** | **OR (95%CI)** | **P** | **OR (95%CI)** | **P** | **OR (95%CI)** | **P** | **OR (95%CI)** | **P** |
| **AST(U/L)** |  | 0.001 |  | 0.001 |  | 0.263 |  | 0.863 |  | 0.304 |
| **Nolmal** | 1.000 |  | 1.000 |  | 1.000 |  | 1.000 |  | 1.000 |  |
| **High** | 1.19(1.08, 1.31) |  | 1.20(1.07, 1.33) |  | 0.88(0.70, 1.10) |  | 0.95(0.50, 1.80) |  | 1.11 (0.91, 1.36) |  |
| **ALT(U/L)** |  | 0.010 |  | 0.009 |  | 0.296 |  | 0.793 |  | 0.054 |
| **Nolmal** | 1.000 |  | 1.000 |  | 1.000 |  | 1.000 |  | 1.000 |  |
| **High** | 0.90 (0.84, 0.98) |  | 0.90(0.83, 0.97) |  | 0.93 (0.81, 1.07) |  | 0.96 (0.73, 1.27) |  | 1.14(0.99, 1.30) |  |
| **Tbil (umol/L)** |  | <0.001 |  | <0.001 |  |  |  | 0.112 |  | 0.626 |
| **Nolmal** | 1.000 |  | 1.000 |  |  |  | 1.000 |  | 1.000 |  |
| **High** | 0.82(0.76, 0.88) |  | 0.88(0.84, 0.93) |  |  |  | 0.78(0.57, 1.06) |  | 0.97 (0.86, 1.09) |  |
| **ALP** |  | 0.035 |  | 0.38 |  |  |  | 0.079 |  | 0.178 |
| **Nolmal** | 1.000 |  | 1.000 |  |  |  | 1.000 |  | 1.000 |  |
| **High** | 0.83 (0.69, 0.99) |  | 0.97 (0.91, 1.04) |  |  |  | 0.75 (0.55, 1.03) |  | 0.74 (0.48, 1.15) |  |
| **GGT** |  | <0.001 |  | <0.001 |  |  |  |  |  | <0.001 |
| **Nolmal** | 1.000 |  | 1.000 |  |  |  |  |  | 1.000 |  |
| **High** | 0.87(0.81, 0.92) |  | 0.83 (0.78, 0.89) |  |  |  |  |  | 1.21(1.09, 1.34) |  |
| **Alb** |  | 0.400 |  | 0.334 |  |  |  | 0.999 |  |  |
| **Middle** | 1.000 |  | 1.000 |  |  |  | 1.000 |  |  |  |
| **High** | 1.24(0.83, 1.84) |  | 0.88 (0.51, 1.52) |  |  |  | 0.000 |  |  |  |
| **Low** | 1.08(0.95, 1.23) |  | 1.13 (0.75, 1.70) |  |  |  | 2.32(0.85, 6.31) |  |  |  |
| **TP** |  | 0.057 |  | 0.511 |  |  |  | 0.257 |  |  |
| **Middle** | 1.000 |  | 1.000 |  |  |  | 1.000 |  |  |  |
| **High** | 0.96(0.66, 1.38) |  | 1.12 (0.78, 1.60) |  |  |  | 0.000(0.000, ...) |  |  |  |
| **Low** | 0.83(0.70, 0.98) |  | 1.13(0.92, 1.38) |  |  |  | 2.32(0.85, 6.31) |  |  |  |
| **G** |  | 0.084 |  | 0.312 |  |  |  | 0.638 |  | 0.035 |
| **Middle** | 1.000 |  | 1.000 |  |  |  | 1.000 |  | 1.000 |  |
| **High** | 0.91 (0.81, 1.03) |  | 0.80 (0.60, 1.07) |  |  |  | 0.91 (0.31, 1.50) |  | 0.85(0.75, 0.96) |  |
| **Low** | 0.90 (0.82, 0.99) |  | 0.85 (0.67, 1.08) |  |  |  | 0.74 (0.37, 1.50) |  | 0.99(0.87, 1.12) |  |

AST: aspartate aminotransferase, ALT: alanine aminotransferase, Tbil: total bilirubin, ALP: alkaline phosphatase, GGT: gamma-glutamyl transferase, TP: total protein, Alb: albumin, G: globulin.
