# Supplementary material for: Liver Function-Related Indicators and Risk of Gallstone Diseases—A Multicenter Study and a Systematic Review and Meta-Analysis
Source: Gastroenterol Res Pract. 2024 Aug 24;2024:9097892. doi: 10.1155/2024/9097892 (PMC11366059; doi:10.1155/2024/9097892)
Supplement: Supporting Information 3 — Supplementary Table S3. Characteristics of the included publications of meta-analysis. [file 9097892.f3.docx]

**Supplementary Table 3. Characteristics of the included publications**

| First Author, Year | Region | Study period | Study Design | Sample size | | \| Risk factors \| \| --- \| \|  \| | NOS |
| --- | --- | --- | --- | --- | --- | --- | --- | --- | --- |
|  |  |  |  | Case group (male/female) | Control group (male/female) |  |  |
| Wei Zheng, 2021 | China | 2018.1-2019.2 | CC | 106(67/39) | 100(58/42) | AST、ALT、GGT 、AKP | 5 |
| Lei Liu, 2017 | China | 2015.10-2016.10 | CC | 128(85/43) | 86(55/31) | ALT、AST、ALP、TP、G、ALB、Tbil | 7 |
| Xutao Chen, 2018 | China | 2015.04-2016.04 | CS | 113(31/82) | 441(267/174) | Tbil | 6 |
| Bo Yang, 2017 | China | 2017 | CS | 45(17/28) | 209(161/48) | ALT、AST | 5 |
| Hui Geng, 2015 | China | 2014 | CC | 100(50/50) | 100(50/50) | ALT、AST | 7 |
| Hongming Xiang, 2020 | China | 2016.05-2018.06 | CS | 925(739/186) | 12799(10074/2725) | ALT、AST、Tbil | 7 |
| Nansng Yu, 2012 | China | 2010.10-2011.08 | CS | 38(20/18) | 777 | ALT、AST、GGT、ALP、TP、ALB、Tbil | 7 |
| Siqi Wang, 2017 | China | 2013.01-2016.12 | CC | 100(0/100) | 100(0/100) | ALT、AST | 7 |
| Quanlin Shan, 2016 | China | 2014.09-2015.09 | CC | 362 | 370 | TBil、 ALT、AST | 6 |
| Jinhui Ding, 2005 | China | 2003-2004 | CS | 412(196/216) | 4972 | ALT、AST | 7 |
| Hanping Qiu, 2013 | China | 2011 | CS | 505(233/272) | 350(156/194) | AST、ALT | 7 |
| Feng Chen, 2003 | China |  | CC | 20 | 75 | AST、ALT | 6 |
| Qianying Luo, 2020 | China | 2019.01-2019.12 | CC | 87(57/30) | 87(61/26) | ALT、AST、GGT、ALP、TP、G、ALB、Tbil | 8 |
| Qing Liu, 2005 | China |  | CC | 60(26/34) | 80(37/43) | ALT、AST、GGT、ALP、TP、ALB、TBil | 6 |
| Tan Ke, 2009 | China | 2007 | CS | 391 | 4398 | ALT、AST | 7 |
| S.-N. Wang, 2006 | China | 2004 | CC | 58(21/37) | 101(55/46) | AST、ALT | 7 |
| Qiyun Gu,2020 | China | 2010.7-2012.12 | CS | 94(56/38) | 2194(1181/1013) | ALT | 7 |
| Hsi-Che Shen, 2014 | China | 2010 | CS | 860(484/376) | 5651(3487/2164) | AST、ALT | 8 |
| Oh-Sung Kwon,2020 | Korean | 2009.01-2018.12 | CS | 394(217/177) | 8514(4479/4035) | AST、ALT、ALP、GGT | 5 |
| Deepak Dhamnetiya, 2018 | India | 2013 | CC | 120(83/37) | 120 | AST、ALT、Tbil | 7 |
| Long Li, 2021 | China | 2017.01-2018.08 | CC | 103(61/42) | 103(66/37) | ALT、ALB、TBil、AST | 6 |
| Chi-Ming Liu, 2006 | China | 2002 | CS | 126(59/67) | 2260(1176/1084) | AST、ALT | 6 |
| Chien-Hua Chen, 2006 | China | 2003.08-2004.04 | CS | 168(74/94) | 3165(1517/1648) | AST、ALT、ALP | 7 |
| Lei Liu, 2021 | China | 2019 | CS | 1017 | 328 | ALT、ALP、TBil、AST、GGT | 6 |
| Zongkui Deng, 2022 | China | 2015.01-2018.12 | CS | 189 | 3404 | AST、ALT | 7 |
| Xinhe Zhang | China | 2016.01-2020.12 | CS | 6537 | 258719 | AST、ALT、ALP、GGT | 6 |
| Biaowei Chen | China | 2019.04-2021.04 | CS | 14 | 84 | GGT、TBil | 5 |
| Jiang Deng | China | 2014-2016 | CC | 1635 | 31569 | ALT、TBil | 6 |

AST: aspartate aminotransferase, ALT: alanine aminotransferase, Tbil: total bilirubin, ALP: alkaline phosphatase, GGT: gamma-glutamyl transferase, TP: total protein, Alb: albumin, G: globulin, CS: cross sectional; CC: case control.
