# Supplementary figures and images for: Liver Function-Related Indicators and Risk of Gallstone Diseases—A Multicenter Study and a Systematic Review and Meta-Analysis
Source: Gastroenterol Res Pract. 2024 Aug 24;2024:9097892. doi: 10.1155/2024/9097892 (PMC11366059; doi:10.1155/2024/9097892)

Supplementary Figure 1. Forest plot of previous studies


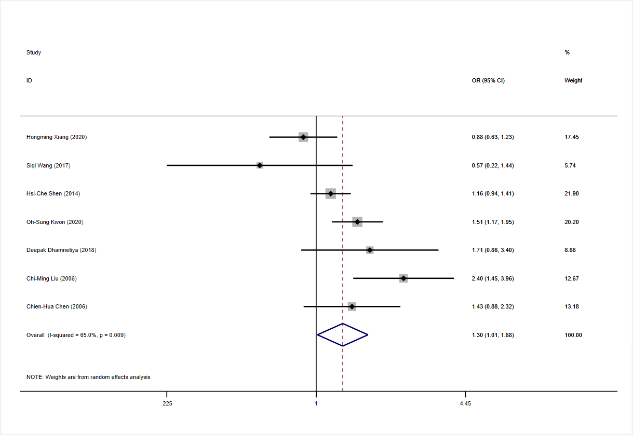

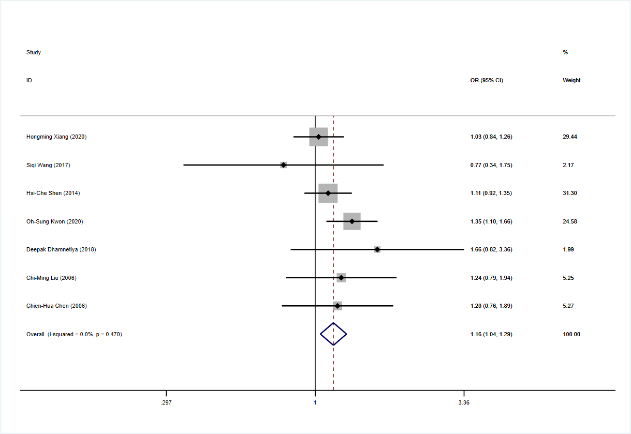


AST

ALT


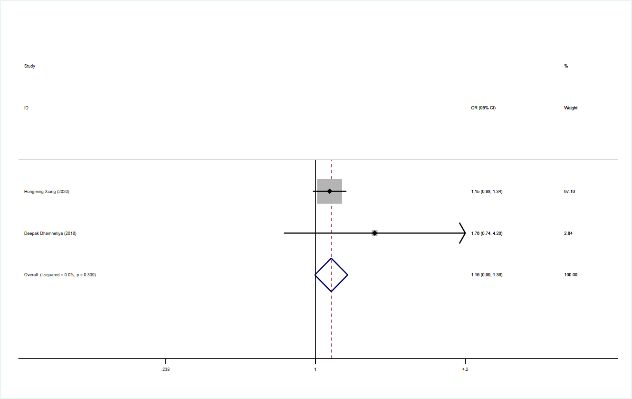

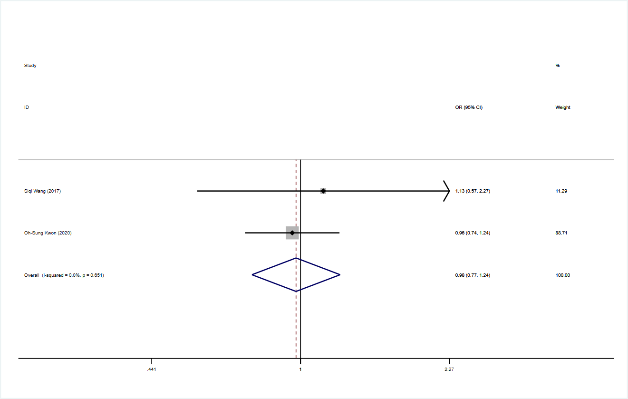


GGT

Tbil


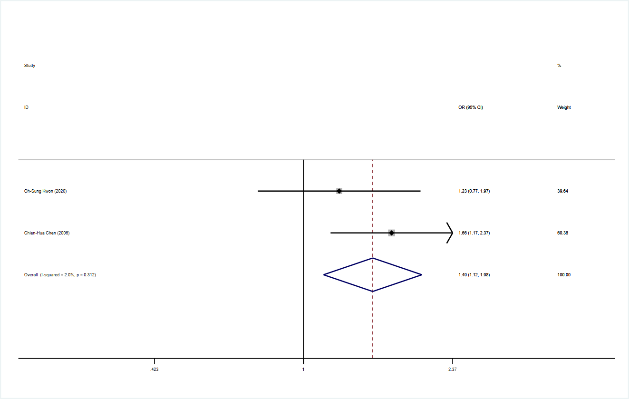


ALP

Supplement: Supporting Information 4 — Supplementary Figure S1. Forest plot of previous studies in meta-analysis. [file 9097892.f4.docx]
