# Supplementary material for: Liver Function-Related Indicators and Risk of Gallstone Diseases—A Multicenter Study and a Systematic Review and Meta-Analysis
Source: Gastroenterol Res Pract. 2024 Aug 24;2024:9097892. doi: 10.1155/2024/9097892 (PMC11366059; doi:10.1155/2024/9097892)
Supplement: Supporting Information 6 — Supplementary Figure S3. Funnel chart of meta-analysis. [file 9097892.f6.docx]

**Supplementary Figure 3. Funnel Chart**


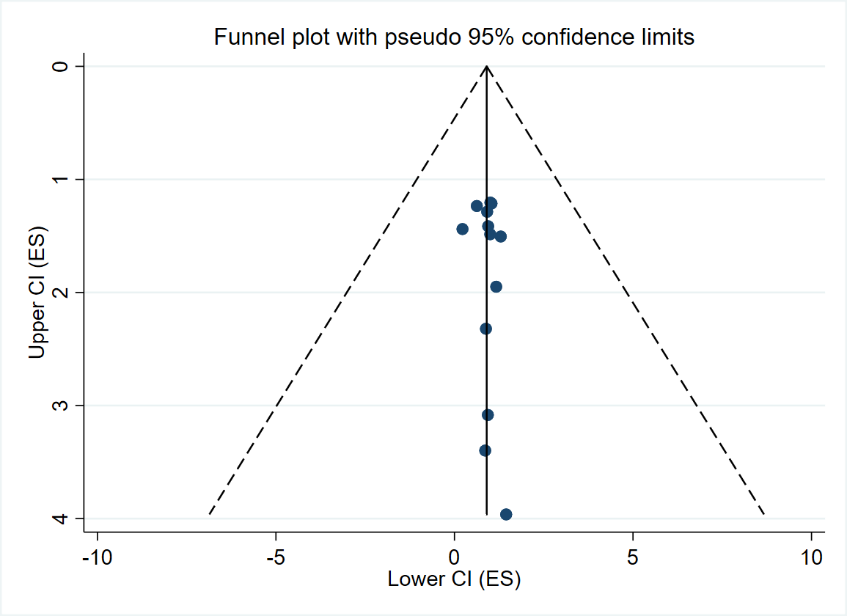

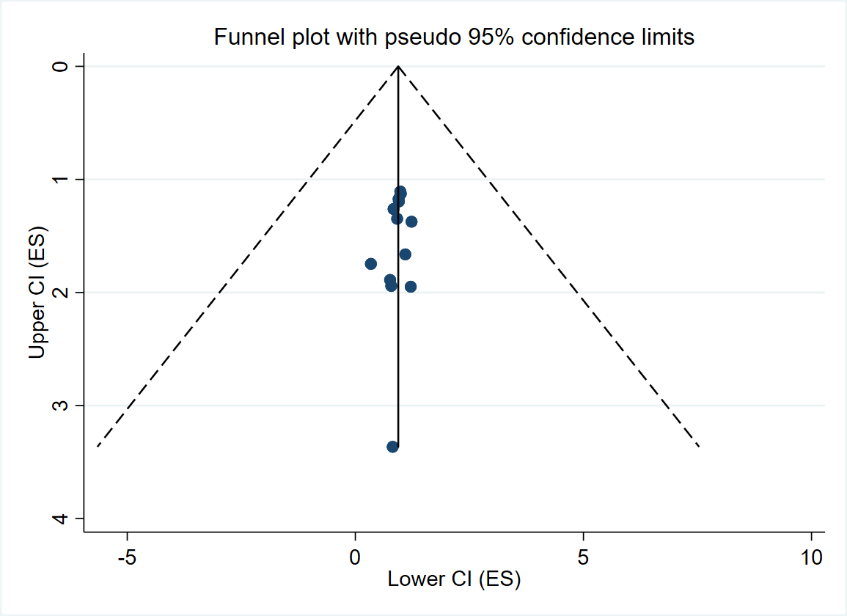


AST

ALT


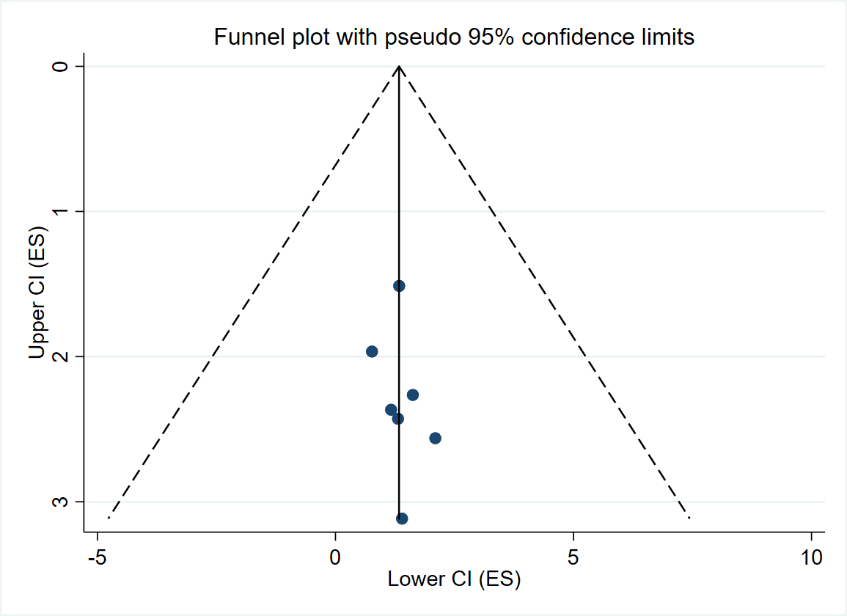

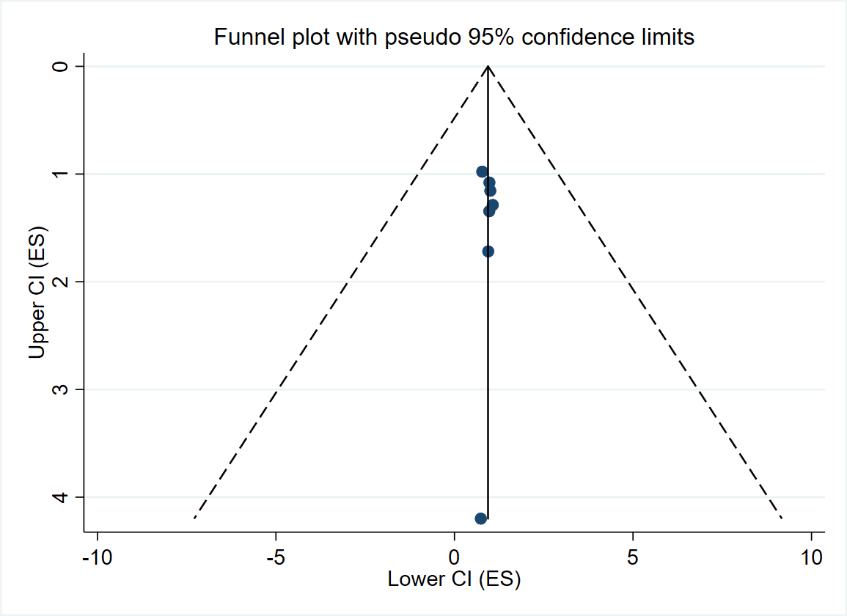


ALP

Tbil


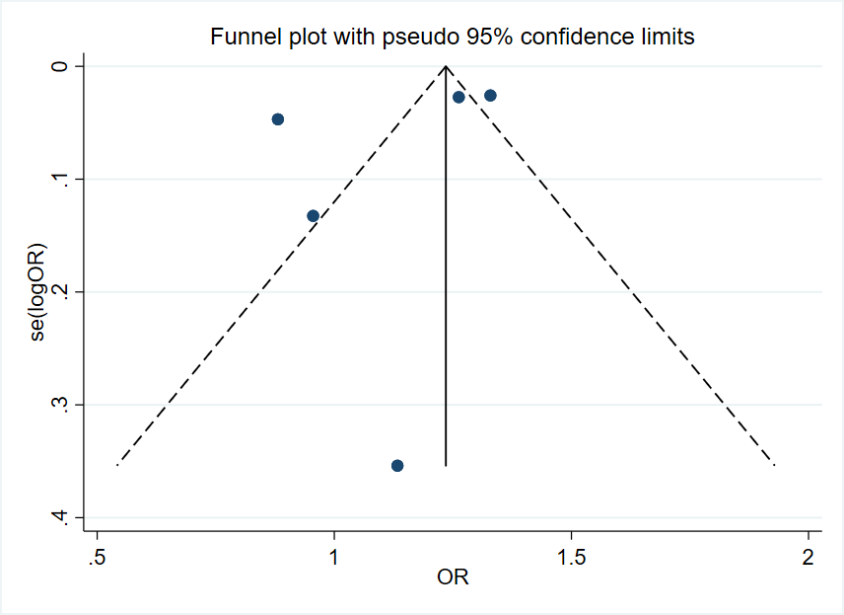

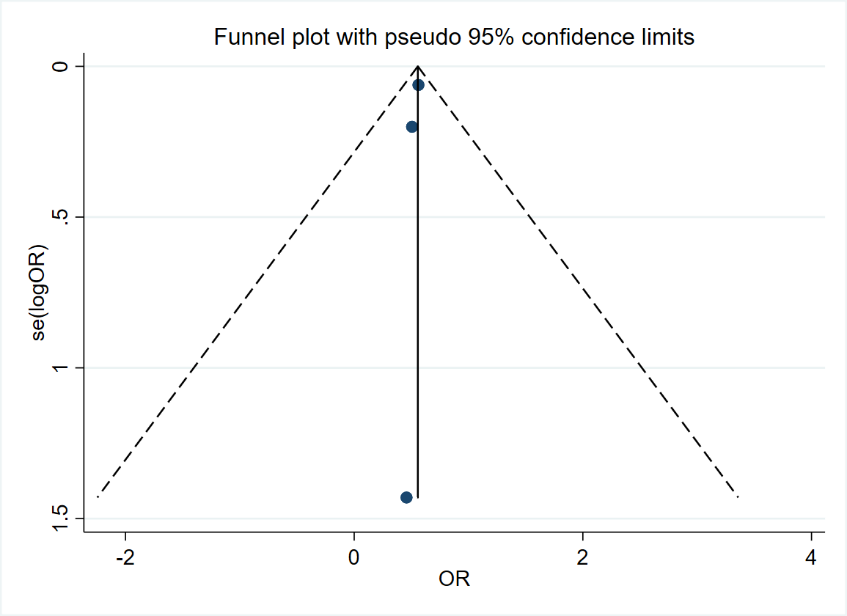


GGT

ALB (H VS M)


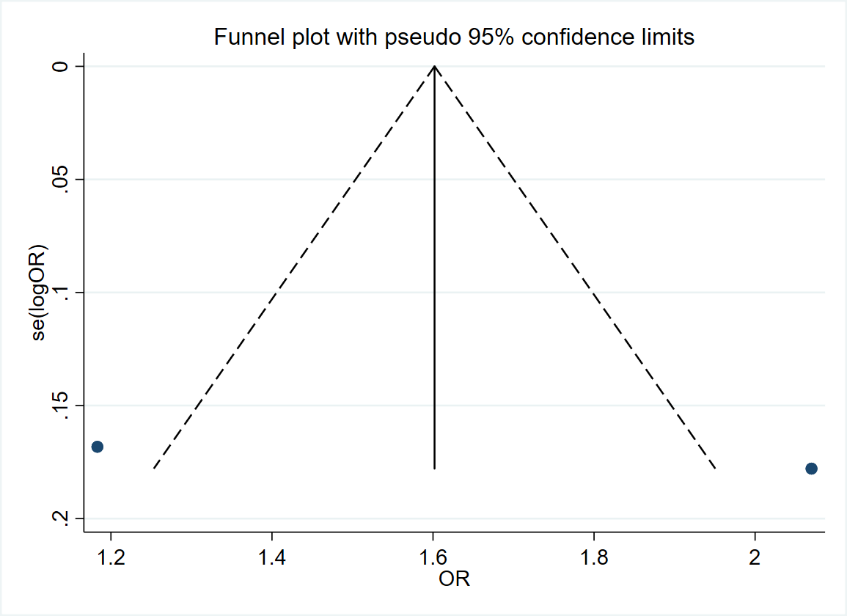

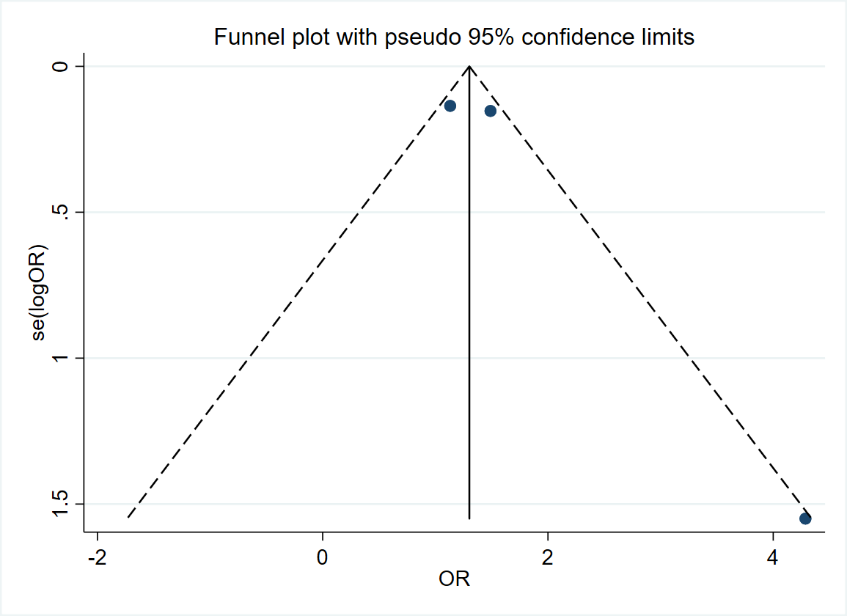


ALB (L VS M)

TP (H VS M)


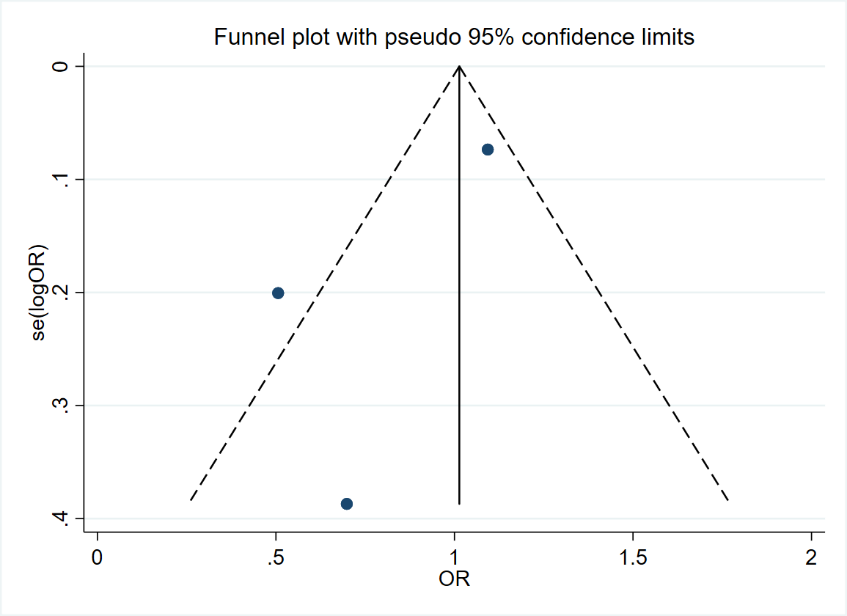

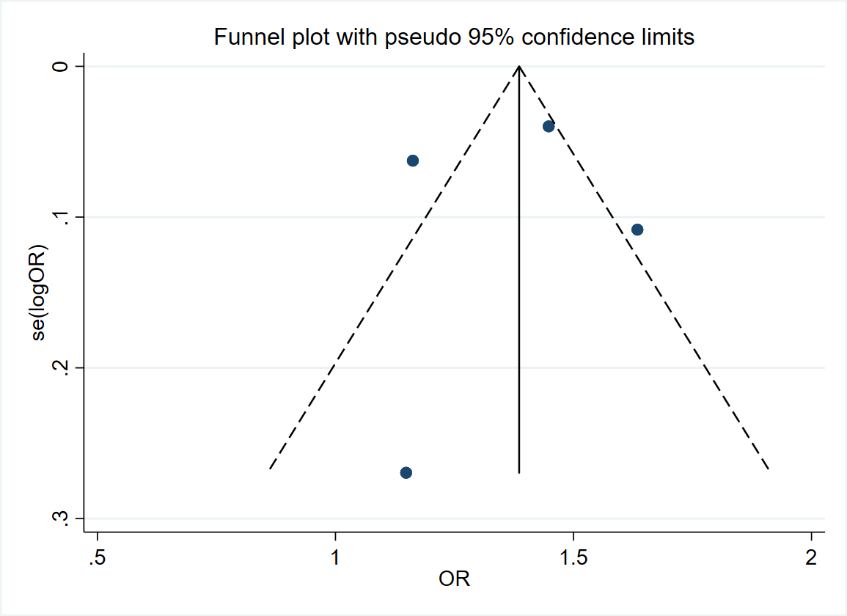


TP (L VS M)

G (H VS M)


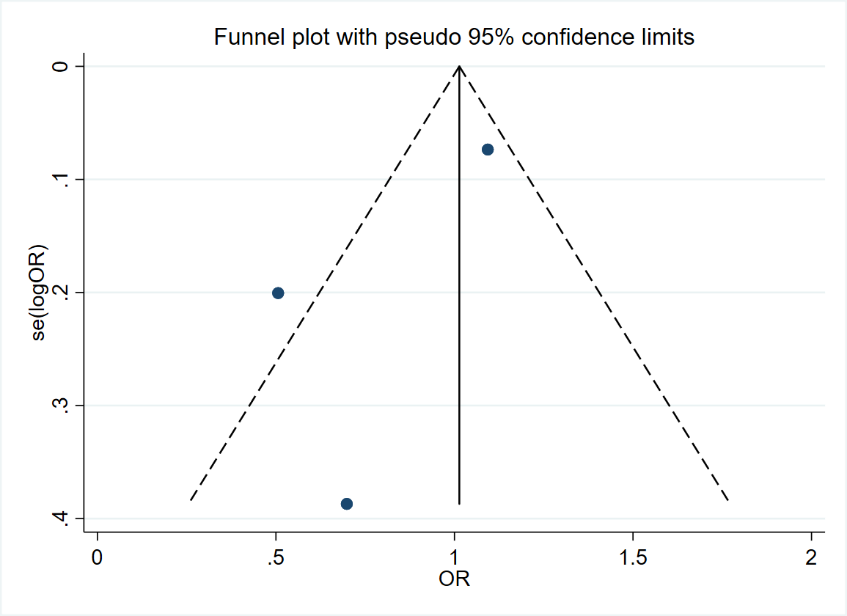


G (L VS M)

AST: aspartate aminotransferase, ALT: alanine aminotransferase, Tbil: total bilirubin, ALP: alkaline phosphatase, GGT: gamma-glutamyl transferase, TP: total protein, Alb: albumin, G: globulin, H vs M: high VS middle, L VS M: Low VS middle.
